# The Effect of Food Unit Sizes and Meal Serving Occasions on Eating Behaviour Characteristics: Within Person Randomised Crossover Studies on Healthy Women

**DOI:** 10.3390/nu10070880

**Published:** 2018-07-08

**Authors:** Billy Langlet, Mona Tang Bach, Dorothy Odegi, Petter Fagerberg, Ioannis Ioakimidis

**Affiliations:** 1 Innovative use of mobile phones to promote physical activity and nutrition across the lifespan (the IMPACT) research group, Department of Biosciences and Nutrition, Karolinska Institutet, 14152 Stockholm, Sweden; petter.fagerberg@ki.se (P.F.); Ioannis.Ioakimidis@ki.se (I.I.); 2 Division of Applied Neuroendocrinology, Department of Neurobiology, Care sciences and Society, Karolinska Institutet, 14152 Stockholm, Sweden; mona.tangbach@gmail.com (M.T.B.); odegidottie@gmail.com (D.O.)

**Keywords:** group ranking, meal duration, food intake, chewing, bites, eating behavior

## Abstract

Manipulating food properties and serving environment during a meal can significantly change food intake at group level. However, the evaluation of the usefulness of such manipulations requires an understanding of individual behavioural changes. Three studies were conducted to explore the effect of unit size and meal occasion on eating behaviour characteristics (food intake, meal duration, number of bites and chews). All studies used a randomised crossover design, with a one-week wash-out period, starting with a familiarisation meal, with the participation of healthy, normal weight females between the ages of 18–35 years. In *Study 1* (*n* = 19) three cube sizes (0.5, 1.0 and 1.5 cm^3^) of vegetable hash and chicken were compared. In *Study 2* (*n* = 18) mashed potatoes and mincemeat were compared to whole potatoes and meatballs. In *Study 3* (*n* = 29) meals served at lunch time (11:00–13:00) were compared to identical meals served at dinner time (17:00–19:00). The largest food unit size lead to significantly increased meal duration in *Study 2* (mean difference 0.9 min, 95% confidence interval (CI) 0.0–1.8), but not in *Study 1* (mean difference 1 min, 95% CI 0.1–2.0). There was a significant increase in number of chews in the large unit size condition of both *Study 1* (mean difference 88, 95% CI 12–158) and *Study 2* (mean difference 95, 95% CI 12–179). Different serving occasions did not significantly change any of the eating behaviours measured. Except for number of bites in *Study 2* (*R^2^* = 0.60), most individuals maintained their eating behaviour relative to the group across unit sizes and serving occasions conditions (*R^2^* > 0.75), which suggests single meal testing can provide information about the behavioural characteristics of individual eating styles under different conditions.

## 1. Introduction

The food intake of humans appears in bursts at discrete time points called meals. In behavioural terms, food intake of single meals is the aggregate of a complex array of eating behaviours, such as bites, chews and pauses [1]. Until recently, methodological limitations caused these behaviours to almost exclusively be studied in laboratory settings [2], which are perceived as main meals (i.e., lunch, dinner) by the participants and may therefore only be relevant to these settings [3]. Currently, the majority of the published literature in this field focuses on the quantification of behavioural differences between differing groups of subjects. For example, existing studies point towards the different eating characteristics between lean and obese [4], male and female [5] and linear and decelerated groups [6]. Other studies use self-reported characteristics (e.g., restrained eating [7]) as grouping parameters, studying the eating behaviour response of these groups to a wide variety of food related cues (e.g., pre-loads [8], stress response [9] and perceived healthiness of the food [10], etc.). Fewer studies, however, focus on individual responses to experimental meal manipulations, often ignoring one of the most important outcomes of such research [11], especially when the study outcomes are considered important for the design of future behavioural interventions [12].

Reliability studies, using both solid and semi-solid food, have shown a high correlation of most eating behaviour parameters under identical conditions in normal weight individuals, which indicates a high relative reliability [6,13,14,15]. However, the inter-individual variability in these studies is usually large, with the standard deviation often exceeding half the size of the mean group value for most of the quantified eating behaviour characteristics. For example, in the study by Laessle & Geiermann, the standard deviation for food intake was >55% of the mean value [14] and in the study by Hubel et al., the standard deviation was >50% of the mean [13].

On a group level, manipulating food properties, the environment and even eating behaviour characteristics, have been shown to cause changes in food intake. For example, liquid foods [16,17], foods with high carbohydrate content [18] and palatable foods [19] appear to increase food intake compared to solid foods, foods rich in protein and bland foods, respectively. On the other hand, eating behaviour characteristics which reduce oral exposure time to the food, such as larger bite sizes [17], shorter oral processing [20,21] and higher eating rate also seem to increase food intake and result in lower satiation [15,22,23]. In turn, shorter oral exposure times have also been associated with obesity in both children [24] and adults [25], usually facilitated through reduced chewing and an overall faster eating rate during the meals.

Another parameter potentially affecting the progression of meals is the unit size of the served food, likely through the modification of the oral exposure time to the foods and increased chewing. For example, increasing the size of the food units seems to induce increased chewing for proper bolus formation [26], linking larger unit sizes to increased chewing across meals (and potentially increased oral processing time) therefore causing a consequent reduction in food intake across meals. On the other hand, manipulating food unit segmentation can also induce the so called “unit bias” leading subjects to consume a set number of food units [27], eventually increasing the overall intake during eating occasions, complicating the study of food unit effect on portion sizes [28]. For example, in a study where unit sizes were manipulated, omelettes, sandwich wraps and pizzas were served in customary food sized units (311, 193 and 555 g) and hors d’oeuvre sized units (13, 24 and 12 g) but no significant difference in food intake was observed [29]. Even though the understanding of the effects of food unit sizes on meals is lacking, it is a very important parameter for the design of realistic lifestyle behavioural interventions, as well as for the facilitation of effective preventive behavioural education in the future [30].

Another important factor for the future design of effective behavioural interventions is the timing of food intake during the day. Specifically, the timing of food intake across the day has been frequently indicated as an important factor influencing energy regulation [31] and consequently the risk of obesity development [32] and the potential effectiveness of weight-loss interventions [33]. While epidemiological studies indicate that the relative size of dinners, compared to lunches, are largely dependent on geographical and cultural criteria [34] (e.g., in Sweden, dinner is usually larger than lunch [35]), there are no controlled studies exploring potential eating behavioural differences caused by serving identical foods at different points of the day. This lack of basic knowledge is often a challenging factor for the design of interventions entailing personalised behavioural feedback during meals (e.g., [36]).

In our present work, our primary goal was to quantify the individual responses of participants from homogenous groups (healthy, normal weight, young adult women) to two external meal manipulations, in order to test the hypothesis that individuals maintain their specific eating characteristics, in relation to the group, despite potential group changes. This hypothesis is tested on two conditions—identical meals served using different food unit sizes and identical meals served at two different time points during a day—which were selected based on their central role in the design of future personalised behavioural interventions and the lack of concrete information about individual and group responses. Thus, normal weight adult females were recruited, with each individual participating in one out of three independent studies. In *Study 1* and *Study 2* participants were presented with identical food items with varying unit size at Swedish lunch time. In *Study 3* participants were presented with identical meals served at different time points during the day (Swedish lunch- and dinner-time). The hypothesis was that although individuals may change their eating behaviour due to different food unit sizes and due to changes in serving occasion (lunch vs. dinner), they will maintain their eating behaviour in relation to other individuals inside the experimental group.

## 2. Materials and Methods 

### 2.1. Experimental Design

**Figure 1:**
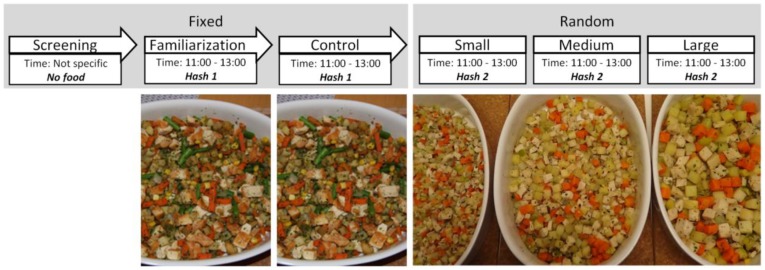
Protocol for *Study 1*, with images of the foods used for each meal session.

**Figure 2:**
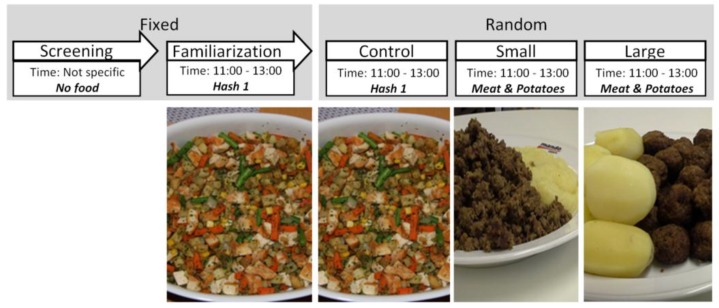
Protocol for *Study 2*, with images of the foods used for each meal session (the tomato sauce served in the meat & potatoes meal is not displayed in the picture).

**Figure 3:**
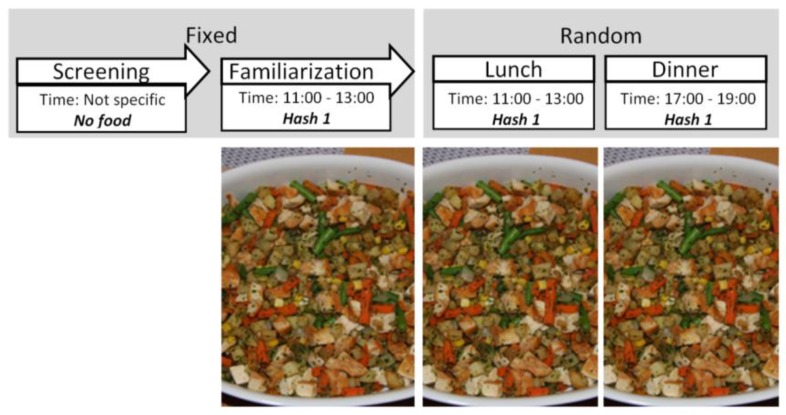
Protocol for *Study 3*, with images of the foods used for each meal session.

Separate participants were recruited for each study and repeated measurements on the same individuals across different conditions were performed in every case. The recruitment process and inclusion criteria were identical across the studies. Pre-study power calculations were performed, identifying a minimum required sample size of 16 for detection of a relevant difference, with α = 0.05 and β = 0.80. *Studies 1* and *2* were designed in order to test the eating behaviour changes across meals with similar food items differing in unit size. *Study 3* was performed to test the effect of identical meals served during different times of the day (i.e., at lunch vs. at dinner). In all the studies, the participants were informed of the study protocols during an introductory informational session, followed by a familiarisation session, during which no data were recorded. Afterwards, control and test sessions followed a crossover design, with test sessions being randomised within each study. Each eating session was followed by a wash-out period of one week before the next session. The specific study protocols are discussed below and can be seen in Figure 1, Figure 2 and Figure 3.

### 2.2. Subjects

All participants were recruited by online and notice-board advertisements at Karolinska Institutet in Stockholm, Sweden. Since our studies are focused on individual responses inside homogenous groups of participants we only recruited healthy, normal-weight females (non-vegetarian, 18–35 years, body mass index (BMI) 18.5–27.0 kg/m^2^). Advertisement respondents attended a recruitment meeting where they were informed about the procedures and the potential risks of the specific protocol, prior to signing the informed consent. Participants were made aware that the studies entailed the collection of eating behaviour information during meals. However, the specific study outcomes (meal intake, duration and number of bites and chews) were not discussed in detail. Afterwards, their weight and height were measured using a BC-418 Segmental Body Composition Analyser (Tanita, Arlington Heights, IL, USA) and a wall mounted stadiometer (Seca, Hamburg, Germany), respectively. Cognitive control of eating (i.e., emotional, external and restrained) was measured using the Dutch Eating Behaviour Questionnaire (DEBQ). However, since the research question was maintenance of eating behaviour within the group, DEBQ results were not used as exclusion criteria. Subjects also completed a questionnaire related to their general health status, their smoking behaviour and potential pregnancies. Based on this, we excluded smokers, pregnant women, participants with previous eating disorder history and individuals who stated strong dislike for the study foods, as well as those having diseases or medications which may affect appetite and meal intake. In total, sixty-six healthy female subjects qualified for participation, with 19 participating in *Study 1*, 18 in *Study 2* and 29 in *Study 3*. Since studies were not running in parallel, the participants were assigned to different studies based on their availability. The per-study recruitment was concluded once the sample size requirements were exceeded. No participant who initially declared interest for their participation in one of the studies was turned down if they fulfilled the inclusion criteria, accounting for eventual differences in the final sample sizes. There was no significant difference in group characteristics across studies (see Table 1). There were no drop-outs in any of the studies. All the presented protocols were approved by the Stockholm Regional Ethics Board (D.nr. 2012/219-31/5, 2014/535-31/3 and 2015/2003-31) and the research procedures were in accordance with the guidelines for human research in the Declaration of Helsinki.

### 2.3. Familiarisation and Control Meals

In all studies, the familiarisation and the control meals were identical and took place during typical Swedish lunch hours (11:00 to 13:00). The served food consisted of pre-cooked vegetable hash with chicken bits (referred to as Hash 1; Findus AB, Bjuv, Sweden), using food identical to that served in other studies [1,37]. The familiarisation meals were not analysed, as they served the purpose of introducing the participants to the research protocols. The control meals were used in order to perform group comparisons across different studies, in order to evaluate potential differences among the recruited samples. The control meals were not part of the primary analysis in *Studies 1* and *2*. In *Study 3*, the control meal (i.e., Lunch) was part of the planned crossover design, as it was compared with an identical meal served during another day as dinner. For macronutrient composition and energy density of all meals, see Table 2.

### 2.4. Unit Size (Study 1)

*Study 1* investigated individual maintenance of eating behaviour ranked across food unit sizes. After familiarisation and control meals, the participants were served three test meals in consecutive sessions, across three weeks in a randomised fashion. The served food (referred to as Hash 2) of the test meals contained a fixed combination of carrots, zucchinis, parsnips, potatoes and chicken (Findus AB, Bjuv, Sweden). The ingredients for the meals were purchased and cooked in-house during a single cooking session. The vegetables and chicken were cut in different cube sizes using a food-dicer (Easy Chopper, Nemco, Hicksville, USA), namely: (i) small unit size (0.5 cm^3^; *Small*) (ii) medium unit size (1.0 cm^3^; *Medium*) and (iii) large unit size (1.5 cm^3^; *Large*), after which they were cooked. The prepared portions were frozen and were later served during the appropriate sessions. All the test meals were served during typical Swedish lunch times, between 11:00 and 13:00.

### 2.5. Unit Size (Study 2)

In *Study 2*, the aim was identical to that of *Study 1*, but the difference in unit size was larger. After the familiarisation meal the participants were served the control meal and two additional test meals in consecutive sessions across three weeks, in a randomised fashion. The served food (Meat & Potatoes) of the test meals contained a fixed combination of minced meat, potatoes and a tomato based sauce (Dolmio, Mars Inc., Mc Lean, VA, USA). For one of the test meals (*Small*), the minced meat was kept as is and the potatoes mashed to a purée using a food processor. For the other test meal (*Large*), the minced meat was shaped into meatballs and the potatoes were kept whole. Again, all the materials were prepared and cooked in a single occasion in-house, then frozen and later served during the appropriate sessions. All the test meals were served during typical Swedish lunch times, between 11:00 and 13:00.

### 2.6. Serving Occasion (Study 3)

*Study 3* investigated individual maintenance of eating behaviour rank across food serving occasions. In all cases, we served Hash 1, identical with the familiarisation and control meals of *Studies 1* and *2*. After the familiarisation meal, the participants ate the same food for lunch (*Lunch*; between 11:00 and 13:00) or for dinner (*Dinner*; between 17:00 and 19:00), in consecutive sessions across two weeks in a randomised fashion. Excluding the serving occasion of the meals, the session procedures were identical.

### 2.7. Session Procedure

Participants were instructed to refrain from moderate and vigorous physical activity 24 hours before each session and to have their breakfast three hours before each session (with the exception of *Study 3*; *Dinner*). Before control and test sessions, participants were supposed to eat the same food for breakfast as the one they reported having eaten before the familiarisation session. Participants were reminded of the session preparations via text message 9:00 the day before each control and test session. The day before the *Dinner* of *Study 3* the message also contained suggestions of what to have for lunch. All the test sessions initiated with weighing of the subjects, after which they were taken to the meal laboratory. There the subjects were presented with the appropriate meal in a ceramic tray next to the plate and were asked to fill in a meal-related questionnaire. In *Study 2*, the tomato-based sauce was presented in a small transparent bowl. Upon completing the questionnaire subjects were informed that they were allowed to eat as much as they wanted for as long as they wanted and add new food to the plate at any time (Ad libitum). They also received information that they were free to leave leftovers on the plate and that they should refrain from doing anything else (e.g., listening to music, using their mobile phones etc.) while in the room. After these instructions, the meal was initiated. Once the participants finished their meal, they completed a similar meal-related questionnaire and the session was over.

### 2.8. Meal Preparation

The day before each session, 1200 g of the appropriate food was moved from the freezer to the refrigerator to thaw. One hour before each scheduled session, a ceramic tray containing the food was placed in an oven at 125 °C. The tray was covered in tinfoil to prevent water evaporation and the food stirred every 20 min to ensure an even temperature. Ten minutes prior to the initiation of the meal the oven was turned down to 70 °C, to ensure the food was an appropriate temperature when served.

### 2.9. Devices

To provide recordings of the weight of food on the plate, a device called the Mandometer^®^ version 4 (Mikrodidakt, Lund, Sweden) was used. The Mandometer^®^ is a portable weighing scale linked to a small computer. The device is used by putting a plate on the scale and then adding food to the plate. The device records weight reduction over time at a sampling rate of 1 Hz, providing raw weight data series in XML format.

In parallel, the meals were videotaped using a digital camcorder (Samsung, Suwon, South Korea). The camcorder was placed 1.5 m away from the subject, angled to capture both activity from the plate and the jaw movements of the subject during chewing.

### 2.10. Questionnaires

The meal-related questionnaires were presented pre- and post-meal. The pre-meal questionnaire started with a free-text question asking participants to recall their food intake since breakfast (or lunch in the case of *Study 3*; *Dinner*) until the initiation of the current study session. The remaining questions prompted participants to rate their hunger and desire to eat on modified 100 mm Visual Analogue Scales (VAS), with five-word descriptors placed along the scale (ranging from “Not at all” to “Extremely”), similar to magnitude scales [38]. The post-meal questionnaire prompted participants to rate their hunger, desire to eat and food liking on similar scales. In our studies, the answers to the meal-related questionnaires were not regarded as outcome variables. Instead, they were used to identify inconsistencies in appetite or the quality and presentation of the food. No meals were excluded based on the collected questionnaire answers.

### 2.11. Data Handling

The video and Mandometer^®^ data were manually transferred to a PC after each session. The meal videos were annotated, using The Observer^®^ XT v12.5 (Noldus Information Technology, Wageningen, the Netherlands), marking six time-stamp events; *meal start*, *meal stop*, *spoonful*, *addition*, *bite* and *chew*, creating one behavioural event log for each eating session. Additionally, the Mandometer^®^ file for each session was analysed only in regards to the cumulative weight changes across the meal, by comparing the initial and the last weight of an individual’s food on the plate, together with in-meal weight changes due to food additions. The output of this analysis is a detailed description of each meal including information about total duration and amount of food consumed, together with detailed information for the occurrence of bites and chews across the session. The data handling procedures followed previously used methods [1,37], focusing on cumulative weight analysis across the meals. 

### 2.12. Statistical Analysis

The presented statistical analyses were done using R 3.2.3 [39]. For box plots the default limit for outliers was used ($1.58\times \mathrm{inter-quartile}\;\mathrm{range}/\sqrt{n}$) described by McGill et al. [40]. Outlier values were not excluded from any of the statistical analyses. A Shapiro-Wilk test and visual inspection of a Q-Q plot and residual vs fitted value plot was made for each outcome variable to ensure the assumption of normality was fulfilled. Food intake, meal duration and number of bites across studies all passed the test for normality and appeared normally distributed upon visual inspection. In *Study 1*, group comparisons of both eating behaviour characteristics and subjective scores among *Small*, *Medium* and *Large* conditions were performed using a linear mixed effects model, due to its advantages over traditional repeated measures data analytics approaches [41]. The linear mixed effects model tests was performed using the lme function of the nlme package [42], followed by a post-hoc Tukey test, to compare the difference between conditions. The test used untransformed values, with session type as fixed effect and the subjects as random effect, with random intercept and fixed slope. In *Studies 2* and *3*, both eating behaviour characteristics and subjective scores between conditions were compared on a group level using dependent *T*-tests. The maintenance of individual eating characteristics across conditions in all the studies were evaluated using Pearson correlations, with medium, high and very high thresholds set at *R^2^* ≥ 0.50, 0.75 and 0.90, respectively [43,44]. The significance threshold of all statistical tests was set at 0.05 and all the values presented in the text are mean (SD), unless otherwise specified. For exact values on mean difference, standard deviation difference, confidence intervals and *p*-values between conditions, see supplementary material “Tables”.

## 3. Results

### 3.1. Food Intake and Meal Duration of Control Meals

**Figure 4:**
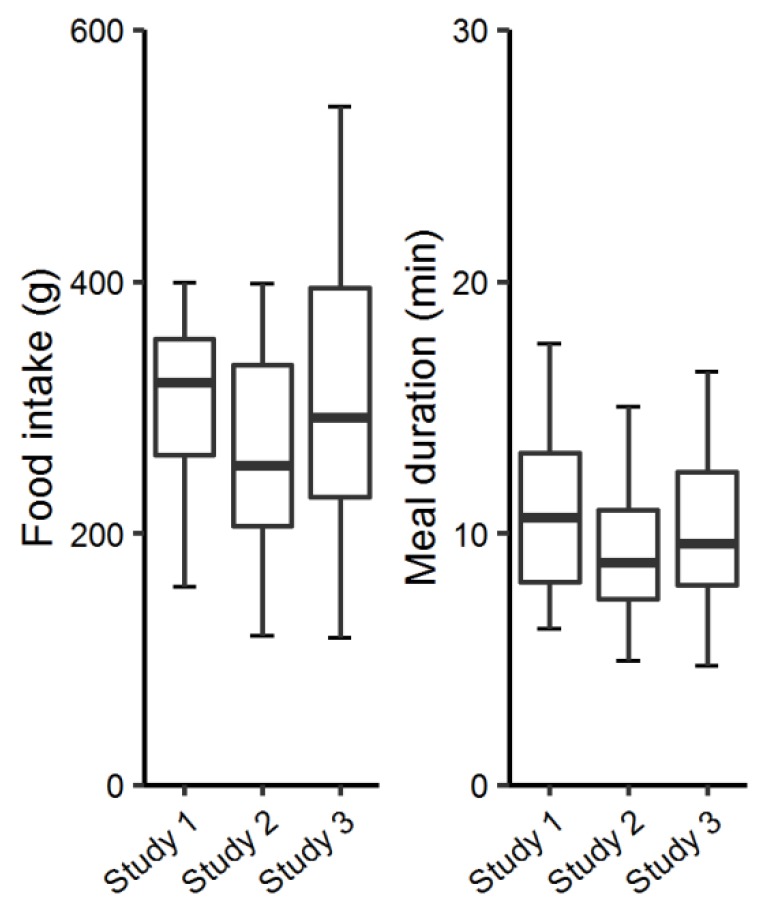
Box plot of food intake and meal duration of the control meals (outliers excluded from the figure).

Figure 4 shows the food intake and the meal duration characteristics of the control meal in all the studies. These meals were used for comparison across studies, since the meal serving occasion and the food type was identical.

### 3.2. Eating Behaviour Characteristics of Test Meals

Table 3 gives an overview of group mean and standard deviation of eating behaviour characteristics in each test meal across studies.

### 3.3. Food Intake

**Figure 5:**
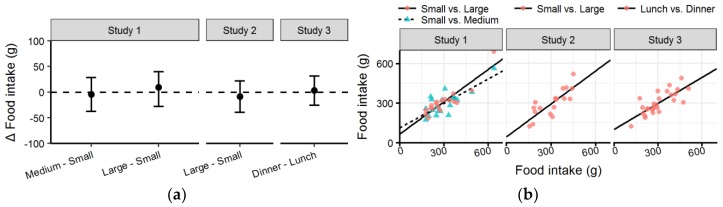
(**a**) Forest plot with mean difference and confidence intervals of food intake between conditions in each study; (**b**) Scatter plot with food intake with the *Small* condition of *Studies 1* and *2* and Lunch of *Study 3* on the x-axis compared to other condition on the y-axis (see legend).

There were no significant differences in food intake across conditions in any of the studies (Table 3 and Figure 5a). The highest mean difference between conditions was 9 g (<5% of mean intake) between the *Small* and *Large* condition in *Study 1*. There was no significant difference in food intake between the *Small* condition in *Study 1* and the *Medium* and *Large* condition (*p* = 0.950 and *p* = 0.900, respectively), nor was there a significant difference between conditions in *Study 2* and *3* (*p* = 0.562 and *p* = 0.819, respectively).

Food intake was highly correlated between all conditions studied (Figure 5b). The correlation of food intake between the *Small* and *Large* condition of *Study 1* was very high (*R^2^* = 0.90). Meanwhile the correlation between *Small* and *Medium* condition of *Study 1*, as well as between conditions in *Studies 2* and *3* were all above the threshold set for high correlation (*R^2^* = 0.78, *R^2^* = 0.81 and *R^2^* = 0.79, respectively).

### 3.4. Meal Duration

**Figure 6:**
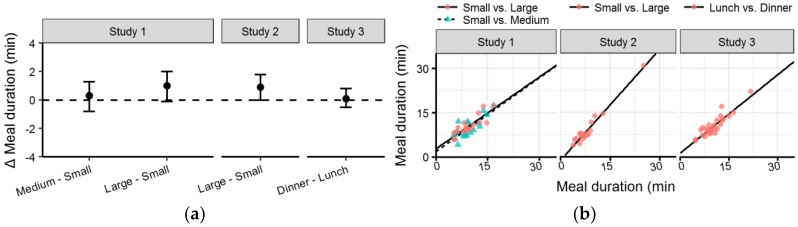
(**a**) Forest plot with mean difference and confidence intervals of meal duration between conditions in each study; (**b**) Scatter plot with meal duration with the *Small* condition of *Studies 1* and *2* and Lunch of *Study 3* on the x-axis compared to other condition on the y-axis (see legend).

In *Study 1* (Table 3 and Figure 6a), there was no significant difference in meal duration between the *Small* condition and the *Medium* and *Large* conditions (*p* = 0.804 and *p* = 0.107, respectively). In *Study 2*, the meal duration in the *Large* condition was significantly longer than in the *Small* condition (*p* = 0.046). In *Study 3*, the mean difference between conditions was very small (0.1 min) and there was no significant difference between conditions (*p* = 0.651).

Meal duration was highly correlated between all conditions studied (Figure 6b).The meal duration of the *Small* and *Large* condition in *Study 2* and the *Lunch* and *Dinner* condition of *Study 3* were very highly correlated (*R^2^* = 0.98 and *R^2^* = 0.90, respectively). Meanwhile, the correlation of the *Small* condition in *Study 1* and both the *Medium* and *Large* condition was high (*R^2^* = 0.81 and *R^2^* = 0.82, respectively).

### 3.5. Bites

**Figure 7:**
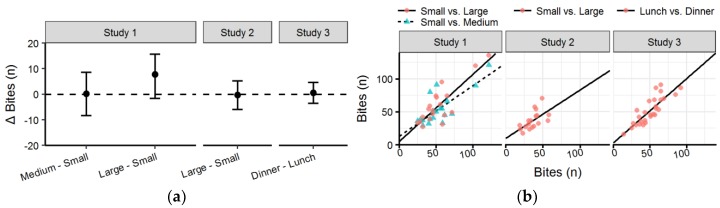
(**a**) Forest plot with mean difference and confidence intervals of number of bites between conditions in each study; (**b**) Scatter plot with number of bites with the *Small* condition of *Studies 1* and *2* and Lunch of *Study 3* on the x-axis compared to other condition on the y-axis (see legend).

There was no significant difference across conditions in the number of bites in any of the studies (Table 3 and Figure 7a). There was no significant difference in number of bites for *Small* condition in *Study 1* in comparison to the *Medium* and *Large* condition (*p* = 0.999 and *p* = 0.132, respectively), nor was there a significant difference between conditions in *Studies 2* and *3* (*p* = 0.918 and *p* = 0.766, respectively).

The number of bites in the *Small* of *Study 1* and the *Medium* and *Large* condition, as well as the *Lunch* and *Dinner* condition in *Study 3* were all highly correlated (*R^2^* = 0.85, *R^2^* = 0.76 and *R^2^* = 0.86, respectively). However, the correlation between *Small* and *Large* in *Study 2* was only moderate (*R^2^* = 0.60).

### 3.6. Chews

**Figure 8:**
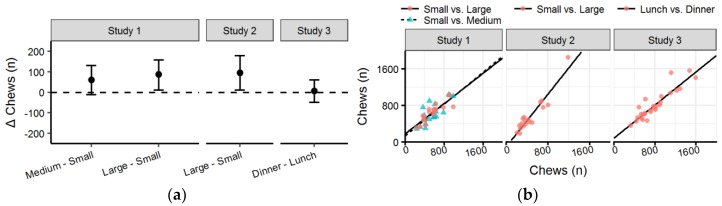
(**a**) Forest plot with mean difference and confidence intervals of number of bites between conditions in each study; (**b**) Scatter plot with number of bites with the *Small* condition of *Studies 1* and *2* and Lunch of *Study 3* on the x-axis compared to other condition on the y-axis (see legend).

In *Study 1* (Table 3 and Figure 8a), there was no significant difference in number of chews between the *Small* and *Medium* condition (*p* = 0.120), but the number of chews was significantly higher in the *Large* condition (*p* = 0.018) compared to *Small*. Similarly, the number of chews in the *Large* condition of *Study 2* was significantly higher than in the *Small* condition (*p* = 0.027). In addition, there was no significant difference in number of chews between *Lunch* and *Dinner* in *Study 3* (*p* = 0.799).

Number of chews was highly correlated between all the conditions in all the studies (Figure 8b). The number of chews in the *Small* and *Large* condition in *Study 2* and *Lunch* and *Dinner* in *Study 3* were both very highly correlated (*R^2^* = 0.94 and *R^2^* = 0.91, respectively). The correlation between the *Small* condition of *Study 1* and the *Medium* and *Large* condition were highly correlated (*R^2^* = 0.78 and *R^2^* = 0.80, respectively).

### 3.7. Subjective Scores

Table 4 shows VAS ratings of the repeated questions in the meal-related questionnaires. Within study comparison across conditions revealed no significant difference in hunger or desire to eat before the study meals. Moreover, no significant difference in hunger, desire to eat or food liking after the meals was observed. Both hunger and desire to eat were significantly higher before, compared with after the meals in all conditions (for *p*-values see supplementary material “Tables”).

## 4. Discussion

Individuals appear to maintain their eating behaviour when eating similar meals under similar conditions on multiple occasions [6,13,14,15]. At the same time, changes in food properties and the environment are known to induce eating behaviour changes on a group level [18,45,46]. Usually, the aim of studies analysing eating behaviour during meals is to support prevention or intervention strategies leading to a reduction of meal intake, with the long-term goal of weight maintenance or restoration [45,46]. However, the individual eating styles of single participants are usually not considered, potentially limiting the usefulness of the reported results when considered in the setting of personalised interventions, due to high within group variability [47].

The current study examined the behavioural responses of individuals, belonging to a homogenous participant group, when the served food unit size and the time point of the meal serving (lunch vs. dinner) were experimentally manipulated. In brief, we hypothesized that although eating behaviour characteristics may be altered as a result of changes in food unit size and serving occasion, individuals will maintain their eating behaviour characteristics relative to the group, pointing towards the existence of individual eating styles. For example, individuals who display a high number of chews under one condition will do so under other conditions and people eating their meals fast in comparison to their peers will remain “fast eaters” despite the overall observed changes in the group behaviour.

On a group level, changing the unit sizes did not significantly affect the food intake in any of the presented studies. However, there appears to be a trend of increased unit sizes resulting in longer meals, with the largest experimentally imposed difference in unit size, i.e., that between the *Small* and *Large* condition in *Study 2*, causing significant meal elongation. Since food intake and number of bites remained stable when food unit size was increased, the observed increase in meal duration in *Study 1* and *Study 2* (12% and 11% increase, respectively) seems to be mediated by an increase in number of chews of the meal (17% and 20% increase, respectively). Meanwhile, lunches and dinners (*Study 3*) did not appear to differ in respect to food intake, meal duration, number of chews and number of bites.

In line with the current report, a previous study reducing the unit size of omelettes, sandwiches and pizzas to less than 1/8 of their customary size, did not cause significant changes in food intake [29]. Similarly, in another study [48], larger units of cucumbers did not elicit increased vegetable intake in children. Contrary to our findings, some past studies observed smaller unit sizes mediating a reduction of total food intake, a phenomenon attributed to the “unit-bias” [27]. For example, in a study reporting serving of chocolate in different unit sizes, normal-weight participants consumed significantly more chocolate when it was served in larger units, with the authors attributing the effect to increased perceived impulsivity of ingesting many units of palatable foods [49]. However, in a more recent study serving cookies, the total food intake of normal-weight females was significantly increased when served larger cookies, with authors attributing the variations in intake on a "segmentation effect" rather than "unit-bias" [28]. In a study where stick-type biscuits were served to participants, increasing the diameter size resulted in a reduced number of bites per gram [50]. This effect may account for some of the discrepancy between unit size studies and illustrate the importance of measuring the number of bites and number of chews in these settings. Additionally, most of the studies in this field only served snack-related food items, and the effect of different unit sizes were not examined across full meals [27,28,48,49,50]. To our knowledge, no studies have investigated the effect of unit size on meal duration, number of bites or number of chews in ad libitum meals.

Regarding group effects on number of chews, several studies have shown that experimentally increasing the number of chews per bite, which can also be regarded as an increase in oral processing time, can lead to an increased meal duration [22,23], which is in line with our findings. The most common way of exploring the effect of chewing and oral processing on intake has been to either instruct participants to adjust their chew frequency, bite sizes etc., or by modifying model foods, changing factors such as texture, hardness and viscosity. For example, in one study model food gels with increased hardness resulted in an increased number of chews (150%) and reduced food intake (29.2%) [51]. More recently unmodified everyday foods have been studied to see if these effects are generalizable. For example, when participants ingested meals with foods that elicited longer oral processing times, they reduced their food intake by 21% [20]. Unlike those studies, in our report, the increased chewing did not result in reduced intake per meal, potentially due to moderate changes in food unit sizes.

In the past, individual timing patterns of food ingestion during a day and their association with the development of obesity have received some attention [31,32], with increased energy intake during the latter parts of the daily cycle interacting with the effects of weight-loss interventions [33]. In practise, information about baseline differences in meal mechanics across the daily cycle is valuable for any intervention which entails behavioural meal advice [37], since baseline eating characteristics should be taken in consideration both in the individualised analysis of eating styles and in the design of personalised eating schedules. However, we are unaware of other studies examining baseline differences across different serving times of the day. Based on our results, it seems that both on a group and on an individual level, the timing of the meals does not affect behaviours during the meals when the meal conditions remain identical. Thus, previously reported epidemiological differences between lunches and dinners [35] can probably be attributed to habitual training [34] and to environmental parameters which affect meals across the day asymmetrically (e.g., [52]).

In regards to the main hypothesis of the current report, on an individual level, the high correlation coefficients across the quantified eating behaviour characteristics indicate maintenance of individual meal behaviours across all conditions, compared to the groups. This seems to be the case for all the quantified parameters and across all the tested conditions, irrespective of mean group changes. Thus, participants who “eat a lot”/“chew a lot”/“eat quickly”/“take many bites” in comparison to their peers in one condition, are likely to do so in other conditions too, no matter if the total behaviour of the group changes or not. However, individual responses differ somewhat in the case of number of bites taken in the meals of *Study 2*, with the correlations between *Small* and *Large* conditions being lower compared to *Studies 1* and *3*. This can potentially be explained by *Study 2* being the only study where food unit sizes in the *Large* condition were big enough to affect individual food handling (i.e., different practices of cutting the food on the plate), which were not controlled for.

In a past study four identical, repeated meals were served to normal-weight, healthy adults, with the tested individuals strongly maintaining their meal characteristics (all correlation coefficients were >0.75). Individuals also maintained their eating behaviour when the served foods differed in regards to energy density and texture among meals, albeit to a lower degree (e.g., food intake correlation was reduced) [15]. Similarly, in the past, we have also reported that normal-weight young adult women maintain their behaviours across identical solid meals (e.g., 0.88 correlation coefficient for food intake across three identical meals) [6], with the outcome repeated in a later study serving semi-solid foods in a controlled environment [14]. These reports (among others [13,53]), support the hypothesis that individuals eating the same food under similar conditions display stable eating behaviours. To our knowledge, no other studies report the maintenance of eating style characteristics when the food unit size (but not food energy density) and meal serving occasion are manipulated.

The results of the present study should be considered with caution when investigating similar effects in real-life settings due to the controlled nature of our protocol. For example, meals in our studies were consumed in a setting lacking the social aspect often present during meals in the real life-setting; between 35% and 40% of main meals in the Nordic countries are consumed in privacy [54]. In addition, our studies only examined the behaviours in healthy, normal-weight young adult women. Future studies would be required to examine if the outcomes remain similar across individuals of different BMIs, different ages and different genders. Additionally, the outcomes of our study might differ across different types of food, especially if more palatable or “indulgent” food items are introduced into the experimental protocol. Finally, the reader should note the potential introduction of behavioural bias due to the information presented to the participants [55], even if a conscious effort not to discuss the specific outcome variables of our studies was made. The main strengths of the studies were that many confounding covariates were controlled through the deployment of a random cross-over design, narrow inclusion criteria, a strict study protocol, a familiarisation meal, a control meal and objective data collection methods.

In summary, our current findings provide evidence that food manipulations which result in an increased number of chews and meal duration do not necessarily lead to a reduced food intake on a group level. Additionally, the present results point towards the persistence of individual behavioural eating characteristics (e.g., “slow eating”, “chewing a lot”, “taking many bites” etc.), despite changes in the food unit sizes and the timing of meals. Clarifying the role of food unit sizes in meals, especially on the level of individual behavioural responses is an important factor for the development of effective screening protocols for the identification of individuals at risk of developing obesity [56], for the realistic design of meal-based behavioural interventions [36] and for the optimisation of preventive behavioural education [30]. For the very same reasons, identifying the baseline behavioural effects of meal timing during the day is important in the development of effective weight-loss interventions based on meal scheduling [31,32]. To achieve the above goals however, additional studies investigating the validity of our outcomes for individuals of different BMIs and ages is required. Future studies should also accommodate individual response analyses methodologies, such as the ones proposed by Hecksteden et al. [57], potentially incorporating repeated measures per individual in all the tested conditions.

## 5. Conclusions

This study provides evidence that big increases in food unit size can lead to meal elongation by increasing the number of chews per meal, but does not always affect the total intake or the number of bites across the meal. Furthermore, identical meals served at different points during the day do not significantly change any of the eating behaviour characteristics on a group level.

More importantly, this report also presents evidence that individuals maintain their eating behaviour relative to their peers when meals are manipulated in regards to food unit sizes or they are served at different points during the day, in controlled environments.

## Figures and Tables

**Table 1:** Group characteristics for the three studies.

	*Study 1* (*n* = 19)	*Study 2* (*n* = 18)	*Study 3* (*n* = 29)
Age, years	22.5 (1.8)	25.9 (4.7)	24.4 (2.7)
Weight, kg	59.1 (5.6)	60.7 (7.8)	60.9 (6.2)
Height, cm	167.3 (4.7)	164.1 (5.4)	164.4 (6.1)
Body mass index, kg/m^2^	21.1 (1.6)	22.5 (2.2)	22.5 (2.0)
Dutch Eating Behaviour Questionnaire			
Emotional, 1–5	2.4 (0.8)	2.1 (0.8)	2.5 (0.8)
External, 1–5	3.3 (0.4)	3.1 (0.4)	3.3 (0.5)
Restrained, 1–5	2.7 (1.0)	2.5 (0.8)	2.4 (0.9)

Values are expressed as mean (SD).

**Table 2:** Macronutrient composition and energy density of foods served.

	Hash 1	Hash 2	Meat & Potatoes
Protein, g/100	9.6	9.1	9.4
Carbohydrate, g/100	8.2	7.8	8.5
Fat, g/100	2.0	2.8	6.8
Energy, kcal (kJ)	91.7 (383.3)	92.8 (387.9)	140.1 (585.6)

**Table 3:** Eating behaviour characteristics across studies and conditions.

	Food Intake (g)	Meal Duration (min)	Bites (*n*)	Chews (*n*)
Study 1—Unit size				
Small	304 (116)	9.4 (3.4)	54.4 (24.4)	566 (212)
Medium	300 (92) ^ns^	9.7 (3.5) ^ns^	54.5 (24.7) ^ns^	626 (238) ^ns^
Large	312 (107) ^ns^	10.5 (3.2) ^ns^	61.9 (29.6) ^ns^	662 (195) *
Study 2—Unit Size				
Small	309 (98)	8.3 (4.7)	36.8 (11.1)	471 (251)
Large	301 (101) ^ns^	9.2 (6.1) *	36.5 (13.6) ^ns^	567 (383) *
Study 3—Meal Occasion				
Lunch	310 (108)	10.2 (3.6)	49.2 (17.9)	802 (316)
Dinner	315 (112) ^ns^	10.4 (3.6) ^ns^	50.8 (19.1) ^ns^	808 (315) ^ns^

Values are expressed as mean (SD). All test statistics refer to within study comparisons. * Significantly different compared to the *Small* condition in *Studies 1* and *2*, or the *Lunch* condition in *Study 3*. ^ns^ not significantly different compared to the *Small* condition in *Studies 1* and *2*, or the *Lunch* condition in *Study 3*.

**Table 4:** Subjective meal scores across studies and conditions.

	Before Meal		After Meal		
	Desire to eat	Hunger	Desire to eat	Hunger	Food liking
*Study 1*					
Small	81.3 (17.8)	82.7 (17.0)	20.4 (20.0) ^a^	12.5 (16.0) ^a^	54.6 (21.6)
Medium	81.6 (21.2) ^ns^	81.6 (19.5) ^ns^	20.8 (19.7) ^ns,a^	12.3 (13.5) ^ns,a^	58.3 (12.5) ^ns^
Large	84.3 (21.8) ^ns^	85.5 (19.7) ^ns^	19.6 (16.2)^ns,a^	9.8 (11.5) ^ns,a^	56.8 (20.9) ^ns^
*Study 2*					
Small	62.7 (25.0)	53.3 (26.9)	10.7 (12.8) ^a^	5.7 (8.1) ^a^	47.4 (21.4)
Large	57.8 (23.4) ^ns^	54.4 (27.8) ^ns^	11.8 (13.9) ^ns,a^	5.8 (7.8) ^ns,a^	48.1 (19.1) ^ns^
*Study 3*					
Lunch	78.2 (16.1)	77.0 (15.5)	15.8 (14.7) ^a^	10.7 (11.2) ^a^	67.8 (17.8)
Dinner	72.8 (17.4) ^ns^	72.9 (16.6) ^ns^	16.5 (14.9) ^ns,a^	8.9 (9.3) ^ns,a^	62.9 (22.1) ^ns^

Values are expressed as mean (SD). ^a^ significant difference to pre-meal scores. ^ns^ not significantly different compared to the *Small* condition in *Studies 1* and *2*, or the Lunch condition in *Study 3*.

## Supplementary Materials

The following are available online at http://www.mdpi.com/2072-6643/10/7/880/s1, Table S1: Group characteristics for the three studies; Table S2: Macronutrient composition of foods served; Table S3: Eating behaviour characteristics of control meals (all studies)—Group characteristics for the three studies; Table S4: Eating behaviour characteristics in all studies; Table S5: Difference in eating behaviour characteristics between conditions in *Study 1*; Table S6: Difference in eating behaviour characteristics between conditions in *Study 2*; Table S7: Difference in eating behaviour characteristics between conditions in *Study* *3*; Table S8: Pearson correlation coefficient (*R^2^*) of meal duration, food intake, bites and chews in all studies; Table S9: Before and after meal questions in all studies; Table S10: Difference in before and after meal ratings between conditions in *Study 1*; Table S11: Difference in before and after meal ratings between conditions in *Study 2*; Table S12: Difference in before and after meal ratings between conditions in *Study 3*; Table S13: Research data.
