# Supplementary material for: The Effect of Food Unit Sizes and Meal Serving Occasions on Eating Behaviour Characteristics: Within Person Randomised Crossover Studies on Healthy Women
Source: Nutrients. 2018 Jul 8;10(7):880. doi: 10.3390/nu10070880 (PMC6073387; doi:10.3390/nu10070880)
Supplement: Supplementary file 1 [file nutrients-10-00880-s001.zip › Table S1 - Tables.pdf]

Supplementary material - Tables

Group characteristics (all studies)

Table 1. Group characteristics for the three studies.

	**Study 1** (n = 19)	**Study 2** (n = 18)	**Study 3** (n = 28)
**Age**, y	22.5 (1.8)	25.9 (4.7)	24.4 (2.7)
**Weight**, kg	59.1 (5.6)	60.7 (7.8)	60.9 (6.2)
**Height**, cm	167.3 (4.7)	164.1 (5.4)	164.4 (6.1)
**BMI**, kg/m^2	21.1 (1.6)	22.5 (2.2)	22.5 (2.0)

Values are expressed as mean (SD)

Macronutrient composition of meals

Table 2. Macronutrient composition of foods served.

	**Hash 1**	**Hash 2**	**Meat & Potatoes**
**Protein**, g/100	9.6	9.1	9.4
**Carbohydrate**, g/100	8.2	7.8	8.5
**Fat**, g/100	2.0	2.8	6.8
**Energy**, kcal (kJ)	91.7 (383.3)	92.8 (387.9)	140.1 (585.6)

Eating behaviour characteristics of control meals (all studies)

Table 3. Group characteristics for the three studies.

	**Study 1**	**Study 2**	**Study 3**
**Food intake**, g	310.8 (79.5)	256.7 (101.8)	310 (108)
**Meal duration**, min	10.9 (3.3)	10.1 (5.9)	10.2 (3.6)
**Bites**, n	57.4 (25.6)	56.9 (24.3)	49.2 (17.9)
**Chews**, n	742.6 (256.2)	765.2 (419.0)	802 (316)

Values are expressed as mean (SD)

Eating behaviour characteristics of test meals (all studies)

Table 4. Eating behaviour characteristics in all studies.

	Study 1			Study 2		Study 3	
	Small	Medium	Large	Small	Large	Lunch	Dinner
**Food intake**, g	304 (116)	300 (92)	312 (107)	309 (98)	301 (101)	310 (108)	315 (112)
**Meal duration**, min	9.4 (3.4)	9.7 (3.5)	10.5 (3.2)	8.3 (4.7)	9.2 (6.1)	10.2 (3.6)	10.4 (3.6)
**Bites**, n	54.4 (24.4)	54.5 (24.7)	61.9 (29.6)	36.8 (11.1)	36.5 (13.6)	49.2 (17.9)	50.8 (19.1)
**Chews**, n	566 (212)	626 (238)	662 (195)	471 (251)	567 (383)	802 (316)	808 (315)

Values are expressed as mean (SD)

Eating behaviour differences

Study 1

Table 5. Difference in eating behaviour characteristics between conditions in study 1.

	Small - Medium				Medium - Large				Small - Large			
	Mean	SD	P	95% CI	Mean	SD	P	95% CI	Mean	SD	P	95% CI
**Food intake**, g	4.3	72.8	0.950	-28.8 - 37.4	-7.2	55.7	0.741	-44.4 - 23.2	-9.4	50.8	0.900	-40.1 - 27.5
**Meal duration**, min	-0.3	2.1	0.804	-1.3 - 0.8	-0.6	2.0	0.339	-1.7 - 0.4	-1.0	1.8	0.107	-2.0 - 0.1
**Bites**, n	-0.2	17.0	0.999	-8.6 - 8.3	-6.2	15.5	0.144	-15.6 - 1.7	-7.8	14.2	0.132	-15.7 - 1.6
**Chews**, n	-60.4	152.8	0.120	-132.2 - 11.5	-18.3	138.2	0.712	-97.8 - 47.7	-88.4	101.7	0.018*	-158.2 - -11.7

Study 2

Table 6. Difference in eating behaviour characteristics between conditions in study 2.

	Small - Large			
	Mean	SD	P	95% CI
**Food intake**, g	8.6	61.7	0.562	-22.1 - 39.3
**Meal duration**, min	-0.9	1.8	0.046*	-1.8 - - 0.0
**Bites**, n	0.3	11.2	0.918	-5.3 - 5.9
**Chews**, n	-95.4	167.8	0.027*	-178.9 - -12.0

Study 3

Table 7. Difference in eating behaviour characteristics between conditions in study 3.

	Lunch - Dinner			
	**Mean**	**SD**	**P**	**95% CI**
**Food intake**, g	-3.2	73.6	0.819	-31.7 - 25.3
**Meal duration**, min	-0.1	1.6	0.651	-0.8 - 0.5
**Bites**, n	-0.6	10.2	0.766	-4.6 - 3.5
**Chews**, n	-6.8	137.7	0.799	-61.3 - 47.7

Association of eating behaviour characteristics

Table 8. Pearson correlation coefficient (R^2) of meal duration, food intake, bites and chews in all studies.

	Study 1			Study 2	Study 3
	**Small - Medium**	**Small - Large**	**Medium - Large**	**Small - Large**	**Lunch - Dinner**
**Food intake**	0.78 (<0.01)	0.90 (<0.01)	0.85 (<0.01)	0.81 (<0.01)	0.79 (<0.01)
**Meal duration**	0.81 (<0.01)	0.86 (<0.01)	0.82 (<0.01)	0.98 (<0.01)	0.90 (<0.01)
**Bites**	0.76 (<0.01)	0.88 (<0.01)	0.85 (<0.01)	0.60 (<0.01)	0.86 (<0.01)
**Chews**	0.78 (<0.01)	0.88 (<0.01)	0.80 (<0.01)	0.94 (<0.01)	0.91 (<0.01)

Values are expressed as R^2 (p-value)

Subjective scores

Table 9. Before and after meal questions in all studies.

	Study 1			Study 2		Study 3	
	**Small**	**Medium**	**Large**	**Small**	**Large**	**Lunch**	**Dinner**
**Before meal**							
Hunger	82.7 (17.0)	81.6 (19.5)	85.5 (19.7)	53.3 (26.9)	54.4 (27.8)	77.0 (15.5)	72.9 (16.6)
Desire to eat	81.3 (17.8)	81.6 (21.2)	84.3 (21.8)	62.7 (25.0)	57.8 (23.4)	78.2 (16.1)	72.8 (17.4)
**After meal**							
Hunger	12.5 (16.0)	12.3 (13.5)	9.8 (11.5)	5.7 (8.1)	5.8 (7.8)	10.7 (11.2)	8.9 (9.3)
Desire to eat	20.4 (20.0)	20.8 (19.7)	19.6 (16.2)	10.7 (12.8)	11.8 (13.9)	15.8 (14.7)	16.5 (14.9)

Food liking	54.6 (21.6)	58.3 (12.5)	56.8 (20.9)	47.4 (21.4)	48.1 (19.1)	67.8 (17.8)	62.9 (22.1)

Values range between 0 and 100 on a Visual Analogue Scale

Subjective score difference

Study 1

Table 10. Difference in before and after meal ratings between conditions in study 1.

	Small - Medium				Medium - Large				Small - Large			
	**Mean**	**SD**	**P**	**95% CI**	**Mean**	**SD**	**P**	**95% CI**	**Mean**	**SD**	**P**	**95% CI**
**Before meal**												
Hunger	1.1	14.7	0.937	-8.6 - 6.4	-4.0	14.0	0.432	-3.6 - 11.7	-3.1	13.4	0.641	-4.7 - 10.6
Desire to eat	-0.3	12.5	0.995	-7.3 - 8.0	-2.9	12.8	0.647	-4.8 - 10.8	-3.4	17.3	0.587	-4.5 - 11.1
**After meal**												
Hunger	0.2	9.3	0.996	-4.7 - 4.3	1.2	6.8	0.879	-5.5 - 3.6	0.6	8.6	0.840	-5.7 - 3.5
Desire to eat	-0.5	11.9	0.989	-7.2 - 8.2	-0.1	16.9	1.000	-7.8 - 7.9	-0.9	13.8	0.988	-7.3 - 8.3
Food liking	-3.7	24.1	0.774	-9.0 - 16.3	0.7	19.7	0.967	-10.5 - 15.2	-2.2	26.6	0.905	-14.2 - 11.5

Values range between 0 and 100 on a Visual Analogue Scale

Study 2

Table 11. Difference in before and after meal ratings between conditions in study 2.

	Small - Large			
	**Mean**	**SD**	**P**	**95% CI**
**Before meal**				
Hunger	-1.1	25.0	0.853	-13.5 - 11.3
Desire to eat	4.9	17.3	0.246	-3.7 - 13.5
**After meal**				
Hunger	-0.1	8.4	0.956	-4.3 - 4.1
Desire to eat	-1.1	11.6	0.660	-6.9 - 4.7
Food liking	-0.7	17.3	0.872	-9.3 - 7.9

Values range between 0 and 100 on a Visual Analogue Scale

Study 3

Table 12. Difference in before and after meal ratings between conditions in study 3.

	Lunch - Dinner			
	**Mean**	**SD**	**P**	**95% CI**
**Before meal**				
Hunger	4.1	17.9	0.239	-2.9 - 11.0
Desire to eat	5.4	14.5	0.060	-0.2 - 11.0
**After meal**				
Hunger	1.8	9.8	0.344	-2.0 - 5.6
Desire to eat	-0.7	15.1	0.814	-6.5 - 5.2
Food liking	5.0	13.0	0.053	-0.1 - 10.0

Values range between 0 and 100 on a Visual Analogue Scale
